# Effective treatment of a child with adenoidal hypertrophy and severe asthma by omalizumab: a case report

**DOI:** 10.1186/s13223-022-00732-9

**Published:** 2022-10-23

**Authors:** Haijing Sui, Huishan Zhang, Wei Ding, Zuotao Zhao, Jiali Mo, Jiexin Yuan, Leping Ye

**Affiliations:** 1 Department of Pediatrics, Peking University First Hospital, No.1 Xi’an Men Street, West District, 100034 Beijing, China; 2 Department of Otorhinolaryngology Head and Neck Surgery, Peking University First Hospital, Beijing, China; 3 Center for Diagnosis & Treatment of United Airways Diseases, Departments of Pediatrics, Otorhinolaryngology Head and Neck Surgery or Dermatology, Peking University First Hospital, Beijing, China; 4 Department of Pediatrics, Shanghai Children’s Medical Center, Shanghai Jiao Tong University School of Medicine, Shanghai, China

**Keywords:** Immunoglobulin E, Allergic rhinitis, Allergic asthma, Obstructive sleep apnea syndrome, Children

## Abstract

**Background:**

Childhood adenoid hypertrophy (AH) is common and is often associated with allergic asthma, resulting in complications like obstructive sleep apnea syndrome (OSAS). Management of the disease and its complications is often challenging.

**Case presentation:**

We report here a case of a 10-year-old boy who suffered from severe allergic asthma and rhinitis and was treated with omalizumab. Before the treatment, the childhood asthma control test (C-ACT, 14), visal analog scale (VAS, 7) and lung function (mild obstructive ventilation dysfunction and moderate to severe dysfunction in ventilation in small airway) were seriously affected. Polysomnography showed OSAS (apnea hypopnea index, AHI, 6.4), low hypooxia saturation (lowest pulse oxygen saturation, LoSpO2, 70%), and adenoid hypertrophy (at grade III). After treating with omalizumab for 4 weeks (once treatment), the ventilation function, symptoms of asthma and allergic rhinitis (C-ACT, 24; VAS, 2), and OSAS (AHI: 1.8 and LoSpO2: 92.6%) were all improved, and the adenoids size was also significantly reduced to grade II. And during the following 3 times of treatment, the allergic symptoms continued improving, and the size of adenoid was reduced to grade I. Even 6.5 months after cessation of omalizumab, the size of adenoid remained at grade I.

**Conclusion:**

This is the first documented case that childhood adenoid hypertrophy can be significantly improved by omalizumab.

## Background

Adenoidal hypertrophy (AH) is a common phenotype in children that is associated with environmental allergies and/or infections. AH often causes obstructions of nasal passages and eustachian tubes, leading to dysfunctional nasal mucus clearance and may result in craniofacial anomalies. AH is also associated with obstructive sleep apnea syndrome (OSAS), [1] which may cause hypoxic injury to various systems and severely affect a patient’s quality of life. A common cause for AH is the reactive proliferation of adenoids resulted from a long term nasal cavity exposure to allergens, such as house dusts mites [2]. This allergic mechanism is similar to the pathogenesis of allergic rhinitis (AR) and allergic asthma, so symptoms may overlap in the same patient.

## Case presentation

A 10-year-old boy (weight 36 kg, BMI 22.5 kg/m2) with AH, severe allergic asthma and perennial AR was admitted to our clinic. At the first visit, the patient demonstrated uncontrolled airway hypersensitivity, with symptoms of frequent nasal obstruction, rhinorrhea, cough, and wheezing. The score of childhood asthma control test (C-ACT) was 14 (not well-controlled) and the score of global evaluation of treatment effectiveness (GETE) was 4 (poorly effective). Pulmonary function testing showed that the patient had mild obstructive ventilation dysfunction and moderate to severe dysfunction in ventilation in small airways, with 77.2% for the forced expiratory volume in the first second in percent predicted values (FEV_1_% pred), 90.5% for forced expiratory volume in the first second/forced vital capacity (FEV_1_/FVC), 54.2% and 37.4% for the forced expiratory flows at 50% and 75% of forced vital capacity (FEF_50_ and FEF_75_), and 49.1% for the maximal mid-expiratory flow (MMEF_75/25_). The total immunoglobulin E (IgE) was 474 kU/L.

According to the *Global Initiative for Asthma (GINA) guidelines* [3], the patient was treated with budesonide/formoterol via turbuhaler (80 µg/4.5 µg, inhalation, twice daily), montelukast sodium tablets (5 mg, oral, daily), cetirizine hydrochloride (10 mg, oral, daily) and mometasone furoate aqueous nasal spray once daily.

After the treatment for more than three months, the symptoms remained uncontrolled, with the unsatisfied pediatric asthma quality of life questionnaire (PAQLQ) score of 121 (there are 23 items totally, the maximum score of a single item is 7 points, score of single item difference ≥ 1.5 points is considered significant, and higher scores mean better quality of life [4]). Because of the urgency to control symptoms as fast as possible, we decided to use omalizumab, an anti-IgE antibody medicine that is effective in controlling various allergies in adult. The drug was used at a dose of 150 mg every four weeks (a dose determined by weight and total IgE level). The patient received 4 times of omalizumab treatment totally.

**Fig. 1:**
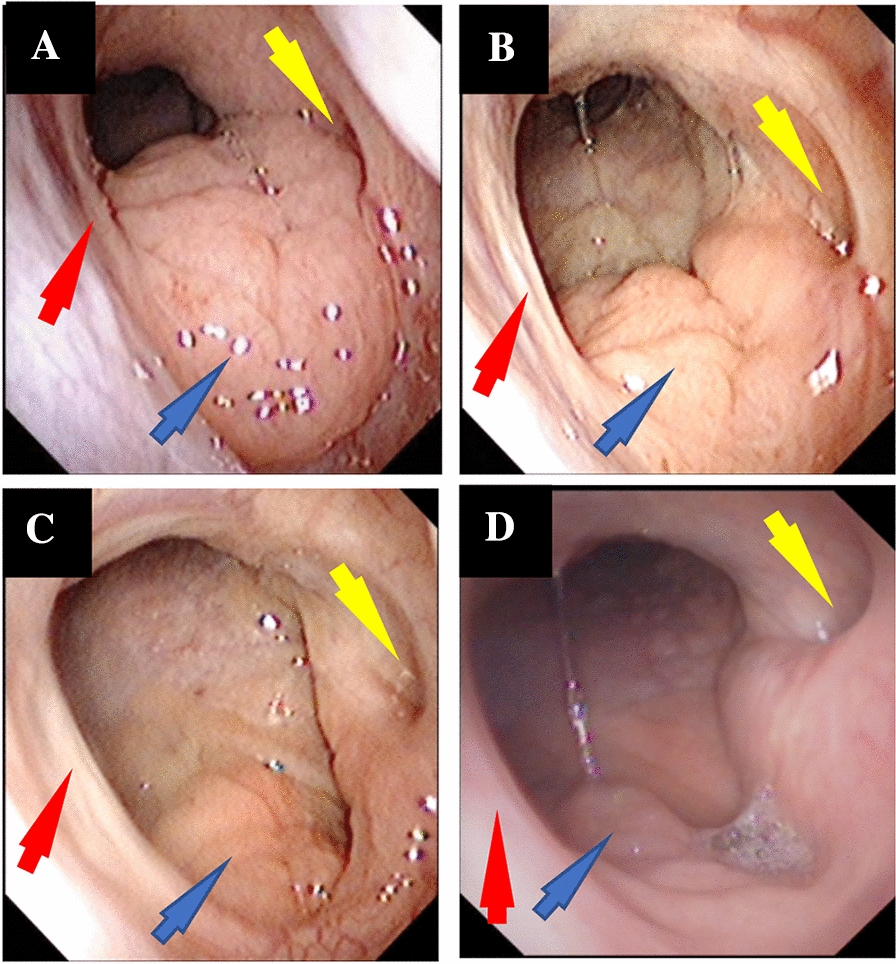
Effect of omalizumab on the adenoidal sizes. ** A** Before treatment. Size shown is rated grade III; **B** After 4 weeks of treatment. The size is rated grade II; **C** After 12 weeks of treatment. The size is now rated grade I; **D** The size was maintained to grade I after treatment ended for 6.5 months. Red arrow: Vomer; Blue arrow: Adenoid; Yellow arrow: Pharyngeal orifice of eustachian tube

The patient was assessed for upper airway inflammation before the treatment with omalizumab. The visual analog scale (VAS) score of allergic symptoms was 7 and the pediatric sleep questionnaire (PSQ) score was 6. Flexible fibro-optic endoscopic examination of the nose and nasopharynx was performed, with AH rated as grade III (Fig. 1A), according to the method of Cassano [5], which contains four grades (with % of choanal obstruction): grade I (0–25%) ; grade II (25–50%); grade III (50–75%) and grade IV (75–100%). In addition, the patient underwent the polysomnography (PSG) to evaluate the effect of AH on quality of sleep and respiration at night. The result showed that obstructive apnea/hypopnea index (OAHI) was 4.3 times/h; the obstructive apnea index (OAI) was 1.3 times/h; and apnea hypopnea index (AHI) was 6.4 times/h. During the process of sleep, the lowest pulse oxygen saturation (LoSpO_2_) was 70%. These results strongly suggested that the child had OSAS.

Four weeks (once treatment) of omalizumab treatment, the symptoms of asthma and AR were relieved significantly, with C-ACT score of 24, PAQLQ 141, VAS 2 and GETE 1 (complete control of asthma). In addition, pulmonary function was also improved dramatically, with FEV_1_% pred 86.3%, FEV_1_/FVC 97%, FEF_50_ 74.6%, FEF_75_ 51.1%, and MMEF_75/25_ 67.1%. Additionally, the size of adenoid tissue went down to grade II (Fig. 1B) after 4 weeks and to grade I after 12 weeks (Fig. 1C). At the same time, the PSQ score was reduced to 2 after 12 weeks, while the OAHI, AHI and OAI decreased to 1.6, 1.8 and 0.3 times/h, respectively, and the LoSpO_2_ increased to 92.6%. Interestingly, the size of adenoids remained at grade I even after cessation of omalizumab treatment for 6.5 months (Fig. 1D), despite stoppage of all intranasal corticosteroids and anti-asthmatic drugs.

## Discussion and conclusions

The size of adenoids increases with age, and normally reaches its maximum by age 6 to 7 years, followed by its regression before adolescence. However, abnormal enlargement or delayed degeneration of adenoid tissues in children is associated with various complications, such as lethargy, poor academic performances, nocturnal enuresis, attention deficit hyperactivity disorder, hypoxemia and so on. At present, adenoidectomy is the definitive method for managing AH worldwide, but it may be associated with a variety of complications, including hemorrhage, infections, palate dysfunction and the risks associated with general anesthesia. In addition, there is still a possibility of recurrence after surgery [6]. It is estimated that 20–30% children with OSAS could have symptoms returned after surgery, about 50% of which were due to adenotonsillar regeneration. [7] While intranasal corticosteroids have also been reported to reduce the size of adenoids, the evidence of long-term efficacy is limited. Moreover, such treatment often requires maintenance therapy, which needs good compliance by parents and children [8]. Therefore, a better alternative treatment is badly needed.

Omalizumab was the first approved anti-IgE drug for the treatment of asthma in the world, with very successful applications in treating allergic diseases in childhood as well as adults. Evidence suggests that omalizumab is not only very effective in reducing the symptoms of asthma itself, but also effective in improving the outcomes of other coexisting conditions, such as AR, allergic conjunctivitis, and atopic dermatitis [9]. In this case, omalizumab systematically improved all the symptoms of asthma and AR, as well as pulmonary function and hypoxemia during sleep. Interestingly, the adenoid size was also reduced significantly. However, the causal relationship between omalizumab and adenoid size is still uncertain, needs further works to confirm. Since the specific and total IgE would be produced locally in the adenoid tissues of atopic children [10], the total serum IgE level of children with AH was significantly increased [11]. Therefore, it is possible that anti-IgE therapy could be effective for AH, just as it is for asthma. Our observation that the size of adenoids remained small after omalizumab was discontinued is significant. As we all know, AR is one of the risk factors of AH [12], We have now followed more than 50 patients with AR and asthma, who had no acute exacerbation of AR after omalizumab treatment, and 30 cases of them were complicated with AH. So, we speculate that the non recurrence of AH may be due to the continuation of effective control of allergic inflammation in the upper airway even after omalizumab treatment.

There are no relevant reports on the treatment of childhood AH with omalizumab to date. This is the first attempt to test the drug for disease in a child. We have found that omalizumab not only improved symptoms, but also reduced the size of adenoids. These observations, if confirmed, could undoubtedly present pediatricians with a new tool in managing children AH and allergic diseases.

## Data Availability

The data that support the findings of this study are available from the corresponding author upon reasonable request.

## Abbreviations

AH: Adenoidal hypertrophy
OSAS: Obstructive sleep apnea syndrome
AR: Allergic rhinitis
ACT: Childhood asthma control test
GETE: Global evaluation of treatment effectiveness
FEV_1_% pred: Forced expiratory volume in the first second in percent predicted values
FEV_1_/FVC: Forced expiratory volume in the first second/forced vital capacity
FEF_50_: Forced expiratory flows at 50% of forced vital capacity
FEF_75_: Forced expiratory flows at 75% of forced vital capacity
MMEF_75/25_: Maximal mid-expiratory flow
IgE: Immunoglobulin E
GINA: Global Initiative for Asthma
PAQLQ: Pediatric asthma quality of life questionnaire
VAS: Visual analog scale
PSQ: Pediatric sleep questionnaire
PSG: Polysomnography
OAHI: Obstructive apnea/hypopnea index
OAI: Obstructive apnea index
AHI: Apnea hypopnea index
LoSpO2: Lowest pulse oxygen saturation
